# Supplementary material for: Transient disome complex formation in native polysomes during ongoing protein synthesis captured by cryo-EM
Source: Nat Commun. 2024 Feb 26;15:1756. doi: 10.1038/s41467-024-46092-3 (PMC10897467; doi:10.1038/s41467-024-46092-3)

Supplementary Information

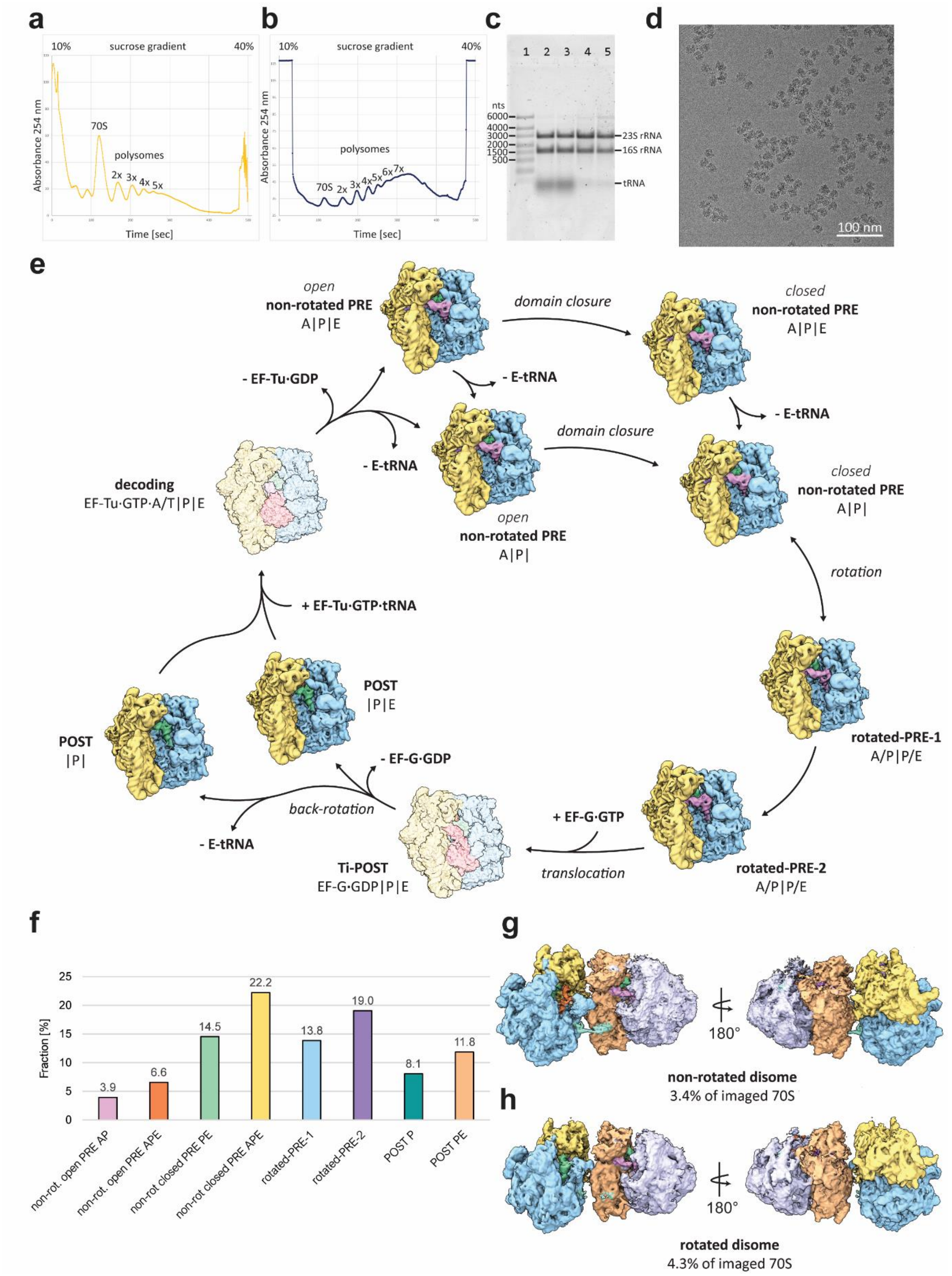

Supplementary Fig. 1: Biochemical and cryo-EM analysis of non-restimulated polysomes. a Sucrose density-gradient analysis of the *E.coli* extract. **b** Final polysomal fraction from size exclusion

chromatography. The most abundant ribosome complex in the extract is the 70S monosome, which is removed by gel filtration from the polysome fraction. **c** RNA gel electrophoresis of *E. coli* extracts (lanes 2, 3) and polysome fractions (lanes 4, 5) with the RNA marker (lane 1) showing the integrity of the 23S and 16S rRNA throughout the purification. **d** Electron micrograph of polysomes after size exclusion chromatography. Scale bar represents 100 nm. **e** Translation cycle showing experimentally observed functional ribosome states isolated from non-restimulated *ex vivo*-derived polysomes. Shown are 30S (yellow), 50S (blue), aminoacyl-tRNAs (A-tRNA, light violet), peptidyl-tRNAs (P-tRNA, green), and exit-tRNAs (E-tRNA, orange). EF-Tu (red) bound decoding (EF-Tu·GDP|A/T|P|E) and EF-G (red) bound translocation (Ti-POST: EF-G·GDP|P|E) intermediates, not observed experimentally, are shown as transparent maps simulated from PDBs 5WFK[19] and 7N2C[21], respectively. All maps were filtered to 5 Å. **f** Distribution of 70S functional states. **g** Disome containing non-rotated $70S_L$ **h** Disome containing rotated $70S_L$. Source data are provided in the Source Data file.

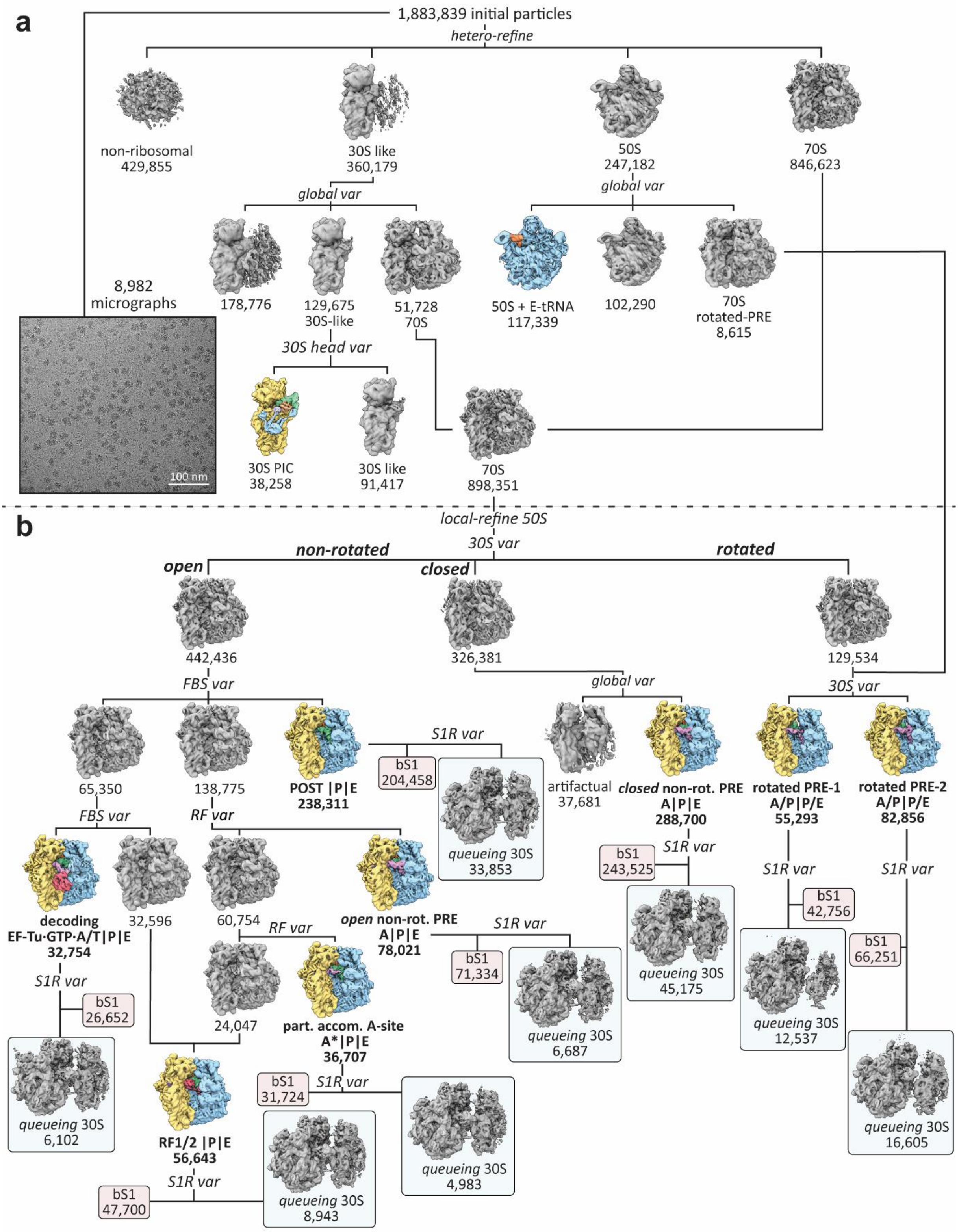

Supplementary Fig. 2: Sorting scheme of ribosomal subunits and 70S particles. Particles were sorted using cryoSPARC v3.3.1[22]. For an initial round of heterogenous refinement **(a)**, 30S, 50S, and 70S 3D templates, as well as a non-ribosomal template were generated internally from the present data using a combination of *ab initio* classification, heterogenous refinement and global 3D variability-based classification[55]. **a** Electron micrograph of polysomes and pre-sorting of particles yielding 30S, 50S and

70S classes (pixel size: 3.18 Å/pixel, box size: 144). Internally generated non-ribosomal, 30S, 50S, and 70S 3D *ab initio* templates were used for an initial round of heterogenous refinement followed by global 3D variability-based classification yielding 30S PIC (pre-initiation complex), 50S and 70S particle populations (*global var* indicates that a global mask was used for classification). **b** Sorting of 70S functional states. Pre-sorted 70S particles were locally refined with a 50S mask. Different local masks were used for subsequent rounds of focused 3D variability-based classification: a mask encompassing the 30S was used for classification focused on the 30S (indicated as *30S var*), a mask encompassing EF-Tu and A/T-tRNA was used for classification focused on the factor binding site (indicated as *FBS var*), a mask encompassing RF1/2 was used for classification focused on the RF1/2 binding site (indicated as *RF var*). Sorted 70S functional states shown in yellow (30S), blue (50S), light violet (A-tRNA), green (P-tRNA), orange (E-tRNA), red (translation factors) were subjected to an additional round of global 3D variability. For all states, global 3D variability indicated further heterogeneity in the region around bS1. A local mask encompassing bS1 and the region around the mRNA exit was used for a final round of focused 3D variability-based classification (indicated as *S1R var*) yielding classes that contained either density for bS1 or a neighboring 30S. Particles exhibiting the additional 30S density were re-extracted at a box size of 234.

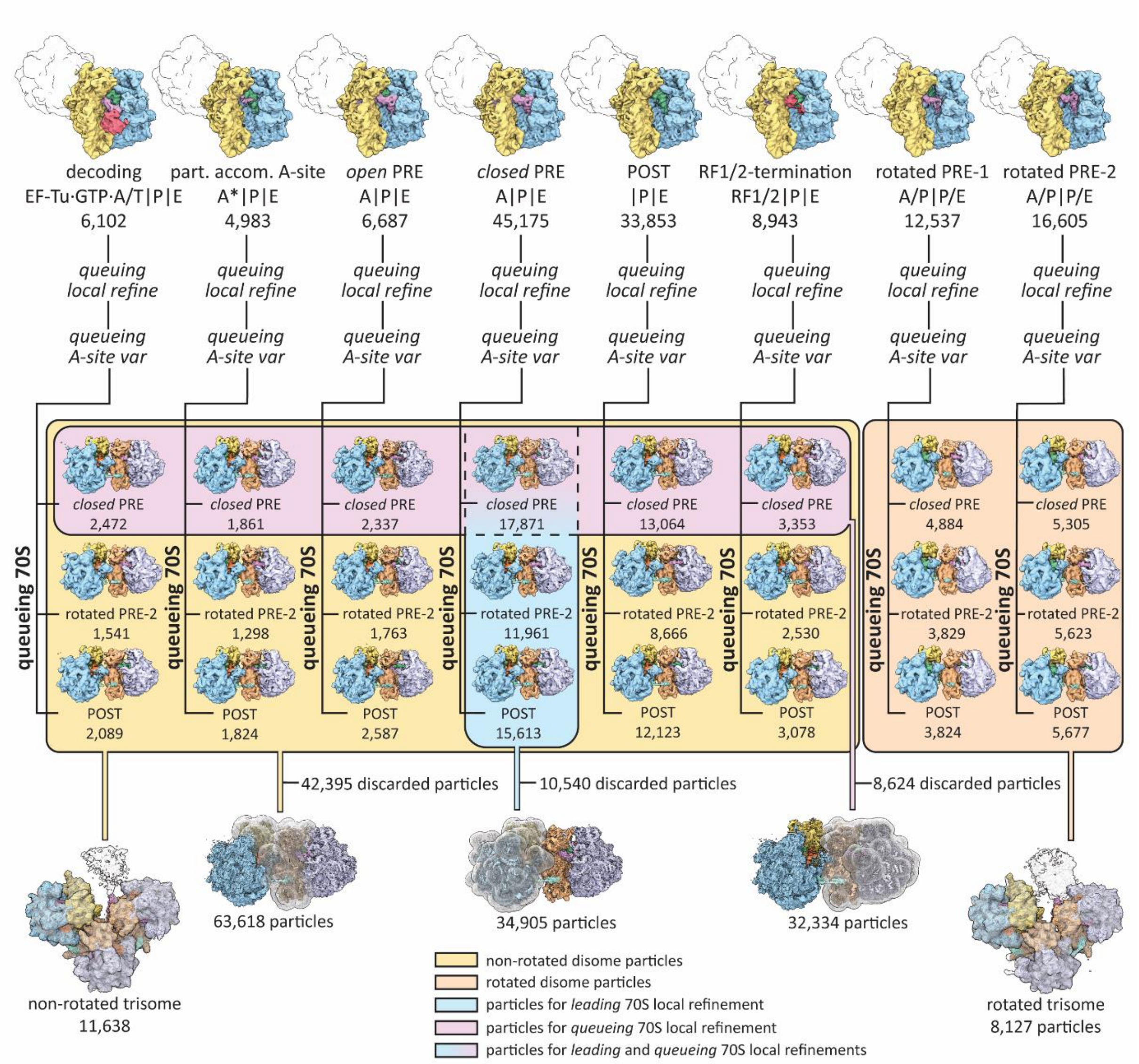

Supplementary Fig. 3: Sorting scheme of disome and trisome particles. Top row: Re-extracted disome particles pre-sorted based on leading 70S functional state shown in yellow (30S), blue (50S), light violet (A-tRNA), green (P-tRNA), orange (E-tRNA), red (translation factors) with unsorted queueing 70S shown as silhouettes. Each of the disome classes was subjected to a local refinement focused on the queueing 70S followed by 3D variability-based classification focused on the *queuing* A-site region. Classification yielded three distinct functional states for queueing ribosomes: closed non-rotated PRE (indicated as closed PRE), rotated PRE, and POST states ($50S_L$ colored in blue, $30S_L$ in yellow, $50S_Q$ in lavender, and $30S_Q$ in peach. $bL9_L$ shown in turquoise). Prior to high resolution refinements of leading 70S, *queuing* 70S, and the disome interface, all closed non-rotated PRE leading 70S particles, all closed non-rotated PRE queueing 70S particles, and all non-rotated disome particles (yellow box) were refined locally (using leading, queueing, and interface masks, respectively) and

subjected to another round of focused 3D variability-based classification. The final particle populations were refined using a pixel size of 1.06 Å/pixel. Non-rotated and rotated trisome complexes were isolated from total non-rotated (yellow box) and rotated (peach box) disome populations using 3D variability-based classification focused on the region downstream of the leading 70S.

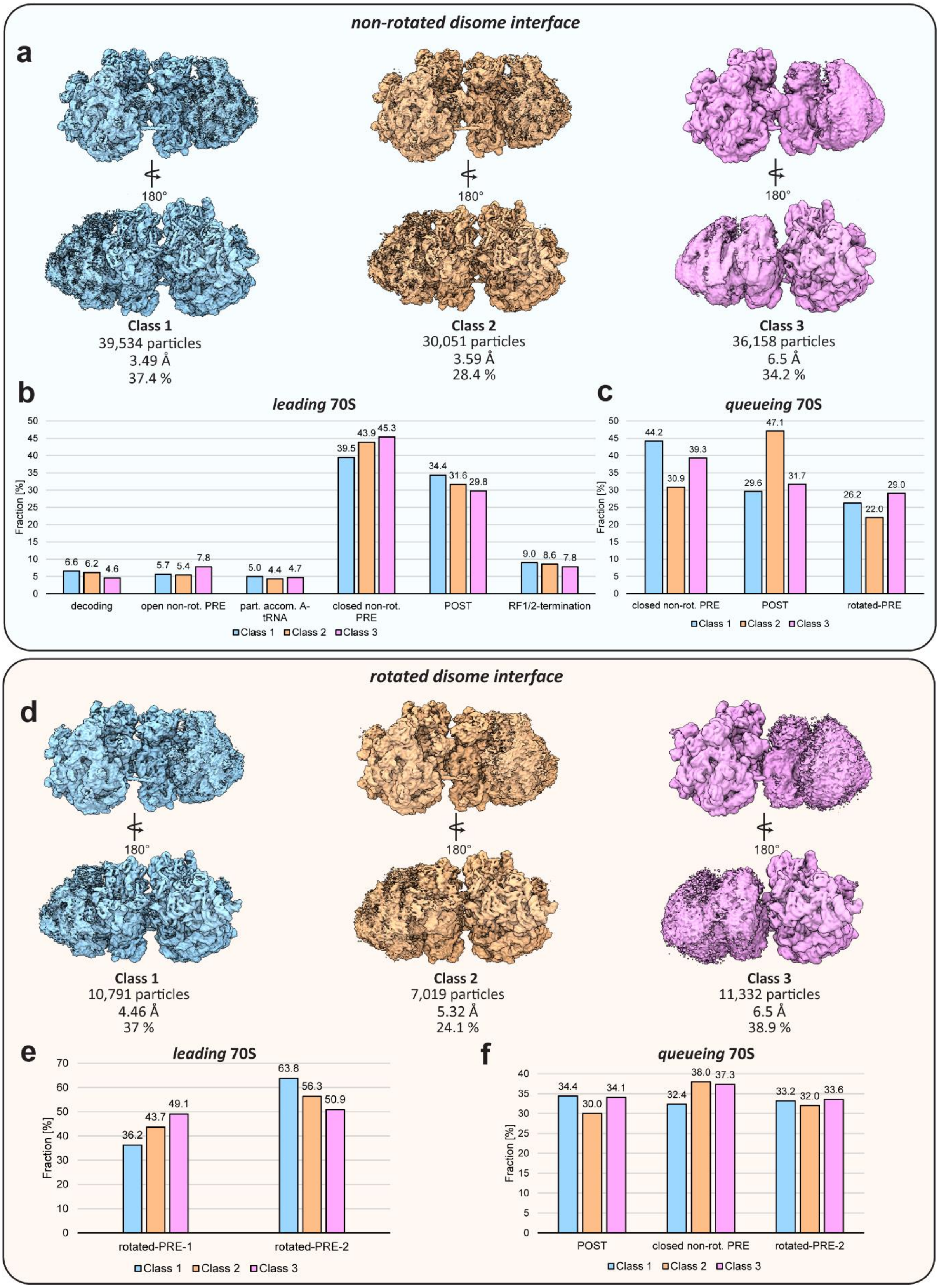

Supplementary Fig. 4: Non-rotated and rotated disome interface classification. **a** non-rotated interface classes isolated by focused 3D variability-based classification[55]. **b and c** per-class functional state distributions of $70S_L$ **(b)** and $70S_Q$ **(c)**. **d** rotated interface classes isolated by focused classification.

e and f per-class functional state distributions of $70S_L$ **(e)** and $70S_Q$ **(f)**. Source data are provided in the Source Data file.

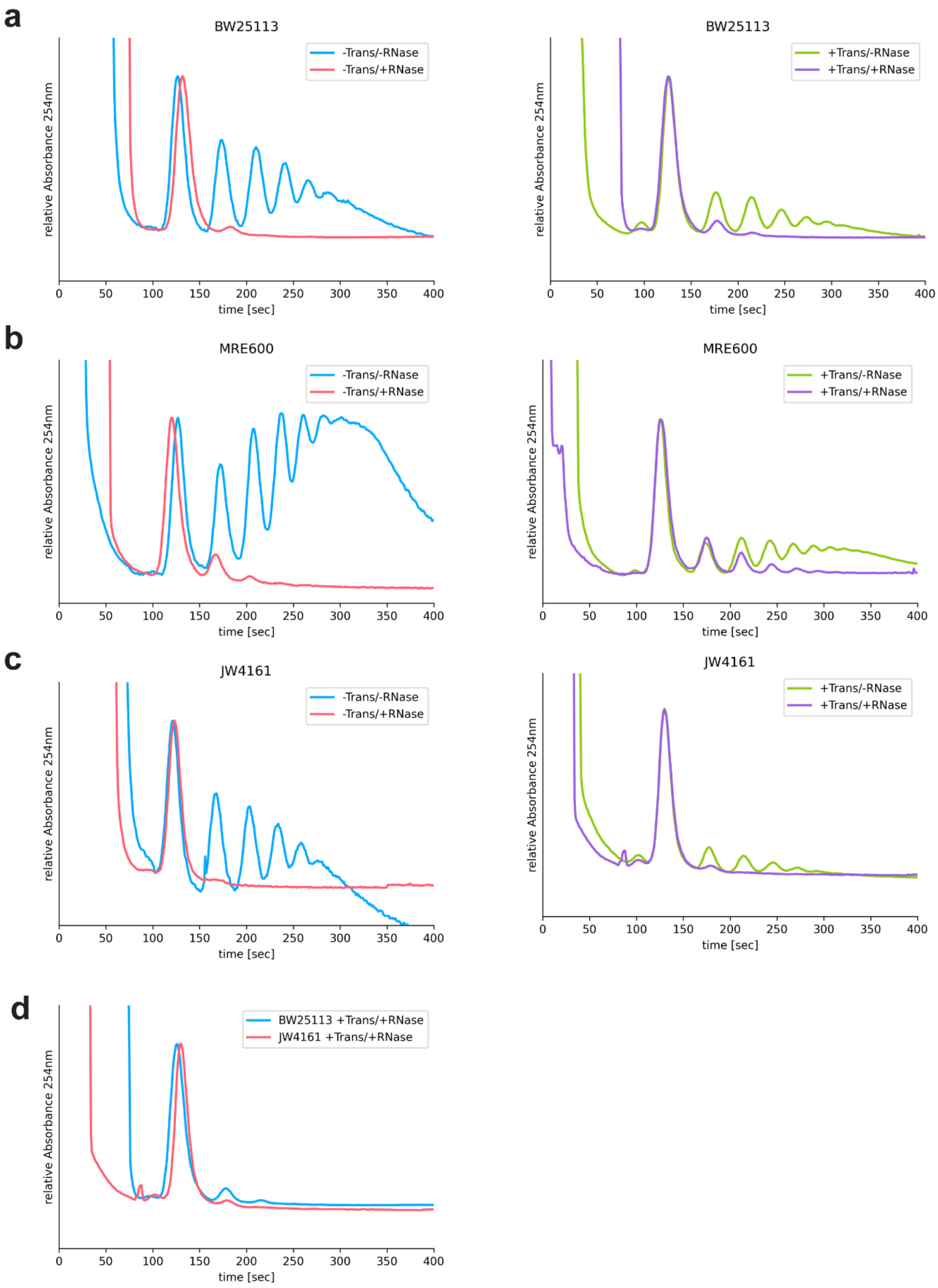

Supplementary Fig. 5: RNase digest experiments of bL9 containing and bL9 lacking polysomes. Normalized sucrose density gradient (SDG) profiles of bL9-containing BW25113 **(a)** and MRE600 **(b)** polysomes and bL9 lacking JW4161 polysomes **(c)**. Left: SDG profiles of non-reactivated polysomes (-Trans) before (blue) and after treatment with 0.25 µg/mL RNase (red). Right: SDG profiles of

reactivated polysomes (+Trans) before (green) and after treatment with 0.25 µg/mL RNase (purple). **d** Overlay of reactivated BW25113 (blue) and JW4161 (red) polysomes after treatment with 0.25 µg/mL RNase. Absorbance values were normalized to the highest monosome peak. Source data are provided in the Source Data file.

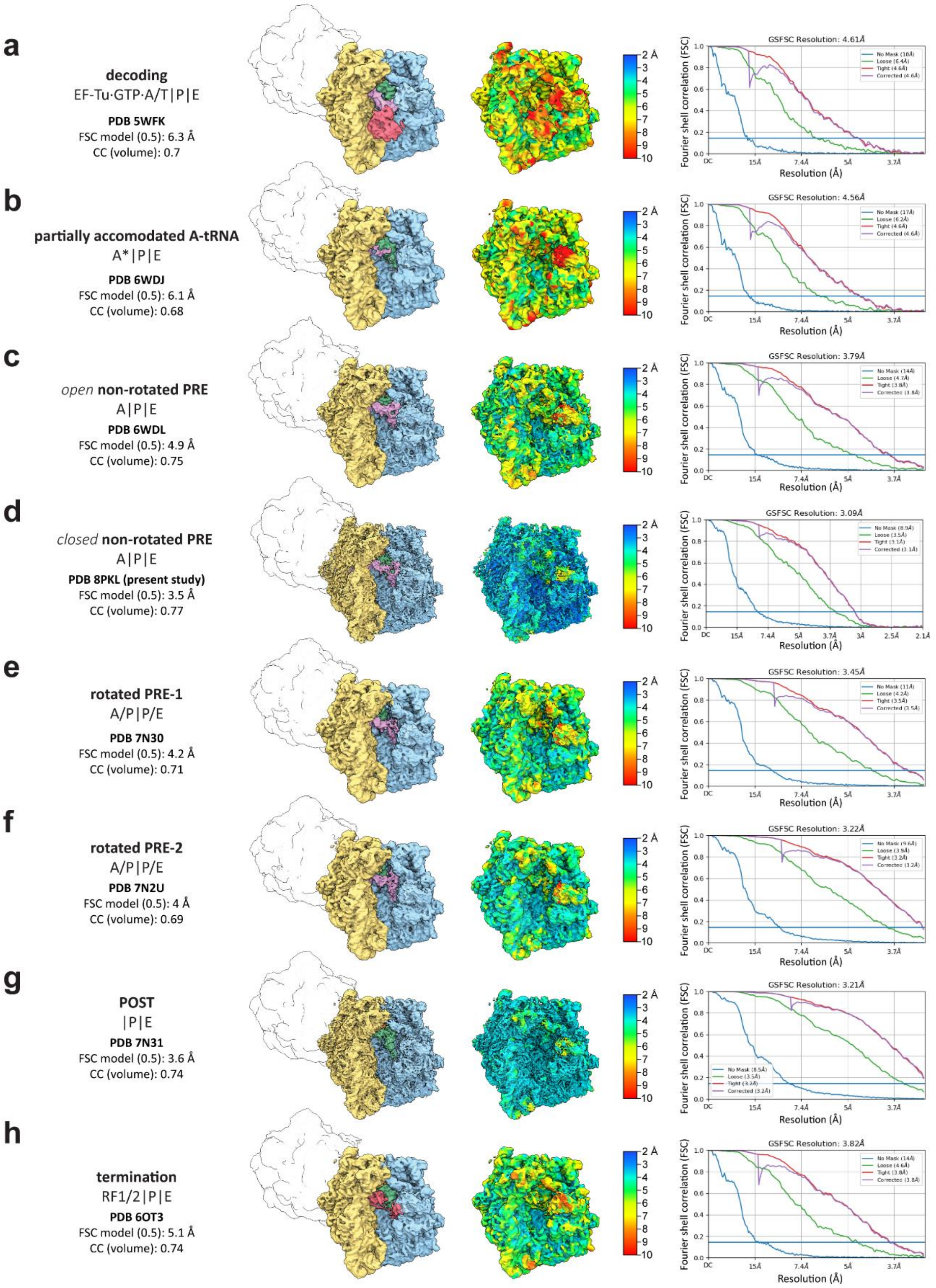

a
decoding
EF-Tu·GTP·A/T|P|E
PDB 5WFK
FSC model (0.5): 6.3 Å
CC (volume): 0.7
GSFSC Resolution: 4.61Å
b
partially accomodated A-tRNA
A*|P|E
PDB 6WDJ
FSC model (0.5): 6.1 Å
CC (volume): 0.68
GSFSC Resolution: 4.56Å
c
open non-rotated PRE
A|P|E
PDB 6WDL
FSC model (0.5): 4.9 Å
CC (volume): 0.75
GSFSC Resolution: 3.79Å
d
closed non-rotated PRE
A|P|E
PDB 8PKL (present study)
FSC model (0.5): 3.5 Å
CC (volume): 0.77
GSFSC Resolution: 3.09Å
e
rotated PRE-1
A/P|P/E
PDB 7N30
FSC model (0.5): 4.2 Å
CC (volume): 0.71
GSFSC Resolution: 3.45Å
f
rotated PRE-2
A/P|P/E
PDB 7N2U
FSC model (0.5): 4 Å
CC (volume): 0.69
GSFSC Resolution: 3.22Å
g
POST
|P|E
PDB 7N31
FSC model (0.5): 3.6 Å
CC (volume): 0.74
GSFSC Resolution: 3.21Å
h
termination
RF1/2|P|E
PDB 6OT3
FSC model (0.5): 5.1 Å
CC (volume): 0.74
GSFSC Resolution: 3.82Å
2 Å
3
4
5
6
7
8
9
10
Fourier shell correlation (FSC)
Resolution (Å)
No Mask
Loose
Tight
Corrected

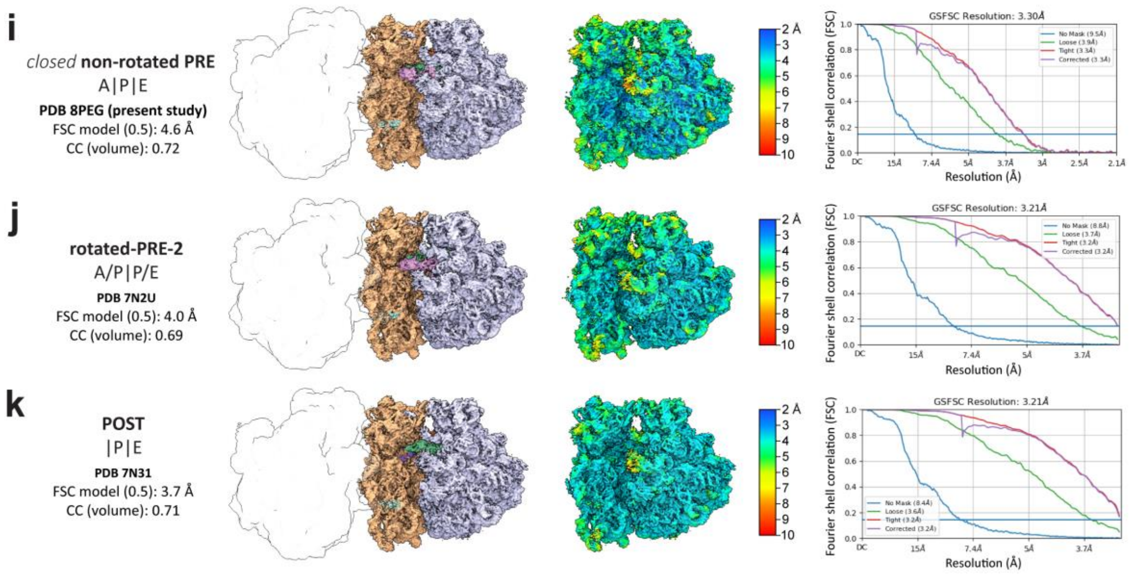

Supplementary Fig. 6: Fourier shell correlation and local resolution of 70S$_L$ and 70S$_Q$ cryo-EM structures. a-k Locally refined maps of 70S$_L$ and 70S$_Q$ functional states. Each row shows from left to right: functional state description and corresponding model information, a map colored by complex components, a local resolution map next to corresponding color key ranging from 2 Å (blue) to 10 Å (red), and gold standard Fourier shell correlation (GSFSC) resolution curve. Resolutions were calculated from half-maps using the FSC cut-off criterion of 0.143. PDB models of known states (a-c, e-h, and j-k) were fitted into experimentally observed maps and cross-resolution (FSC model) as well as cross-correlation (CC) were calculated. Functional states colored in yellow (30S$_L$), blue (50S$_L$), peach (30S$_Q$), lavender (50S$_Q$), light violet (A-tRNA), green (P-tRNA), orange (E-tRNA), and red (translation factors). Disome complex regions excluded by the refinement masks are indicated as silhouettes.

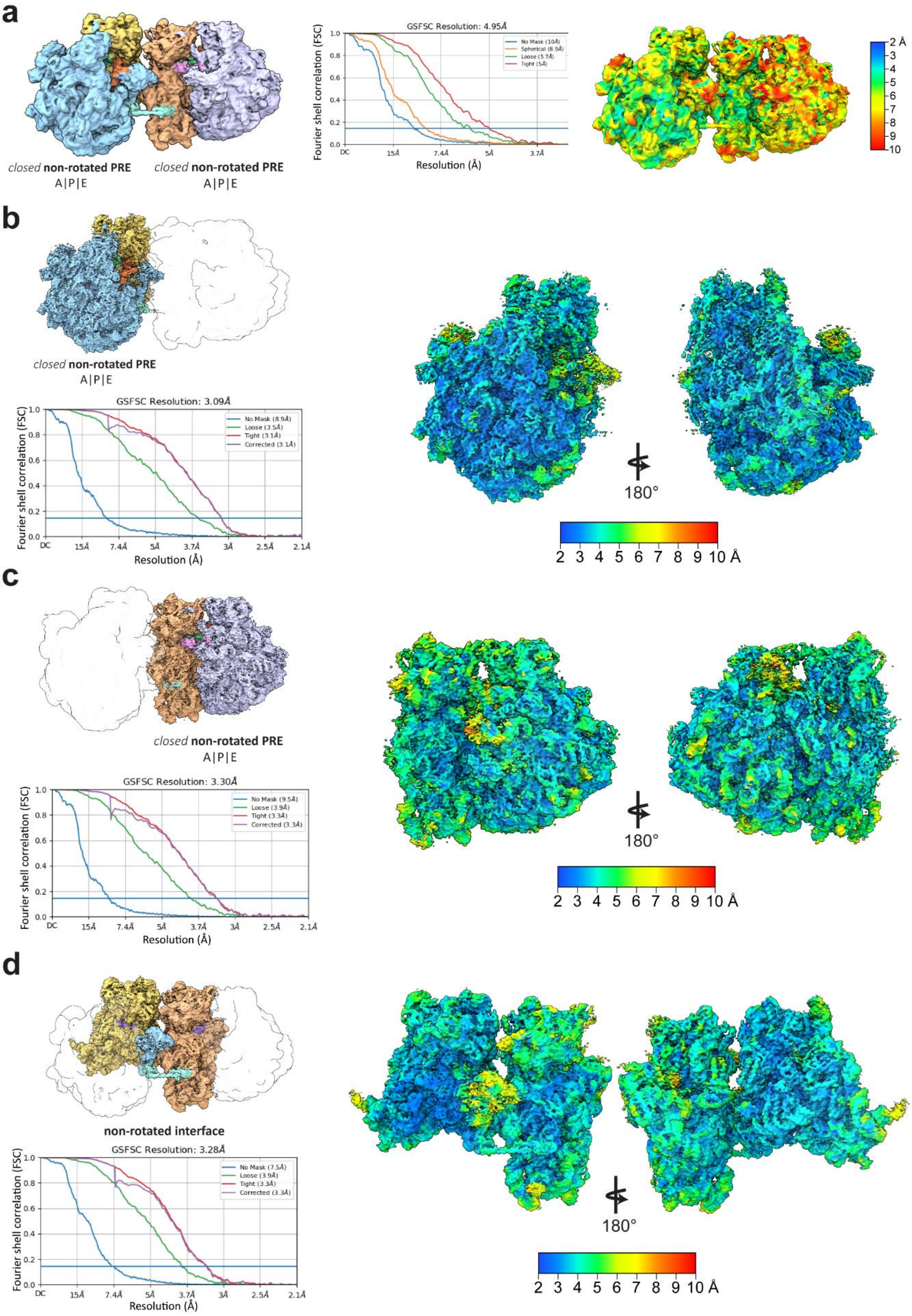

a
closed non-rotated PRE
A|P|E
closed non-rotated PRE
A|P|E
GSFSC Resolution: 4.95Å
No Mask (10Å)
Spherical (8.9Å)
Loose (5.7Å)
Tight (5Å)
Fourier shell correlation (FSC)
Resolution (Å)
2 Å
10
b
closed non-rotated PRE
A|P|E
GSFSC Resolution: 3.09Å
No Mask (8.9Å)
Loose (3.5Å)
Tight (3.1Å)
Corrected (3.1Å)
Fourier shell correlation (FSC)
Resolution (Å)
180°
2 3 4 5 6 7 8 9 10 Å
c
closed non-rotated PRE
A|P|E
GSFSC Resolution: 3.30Å
No Mask (9.5Å)
Loose (3.9Å)
Tight (3.3Å)
Corrected (3.3Å)
Fourier shell correlation (FSC)
Resolution (Å)
180°
2 3 4 5 6 7 8 9 10 Å
d
non-rotated interface
GSFSC Resolution: 3.28Å
No Mask (7.5Å)
Loose (3.9Å)
Tight (3.3Å)
Corrected (3.3Å)
Fourier shell correlation (FSC)
Resolution (Å)
180°
2 3 4 5 6 7 8 9 10 Å

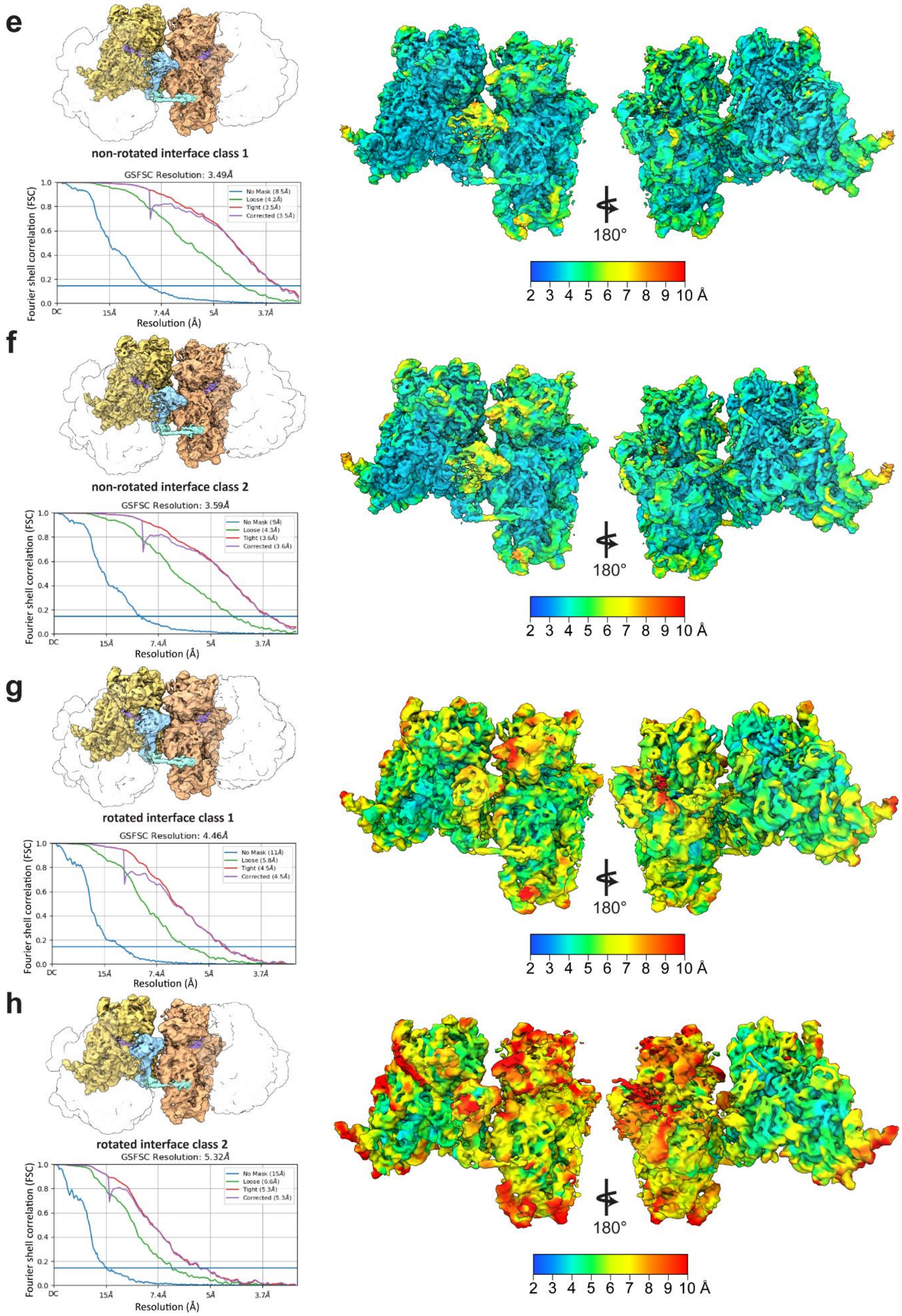

Supplementary Fig. 7: Fourier shell correlation and local resolution of elongating disome and disome interface cryo-EM structures. a Globally refined map, gold standard Fourier shell correlation

(GSFSC) resolution curve, and local resolution map of the disome complex containing $70S_L$ and $70S_Q$ in closed non-rotated PRE states. **b-h** Locally refined maps of $70S_L$ **(b)** and $70S_Q$ **(c)** in closed non-rotated PRE states, non-rotated disome interface **(d)**, and interface classes **(e-h)** yielded by focused 3D variability-based classification[55]. Each row shows a local map colored by complex components with state description (top) and gold standard Fourier shell correlation (GSFSC) resolution curve (bottom) next to a local resolution map with corresponding color key ranging from 2 Å (blue) to 10 Å (red). Resolutions were calculated from half-maps using the FSC cut-off criterion of 0.143. Functional states colored in yellow ($30S_L$), blue ($50S_L$), peach ($30S_Q$), lavender ($50S_Q$), light violet (A-tRNA), green (P-tRNA), orange (E-tRNA), and red (translation factors). Disome complex regions excluded by the refinement masks are indicated as silhouettes.

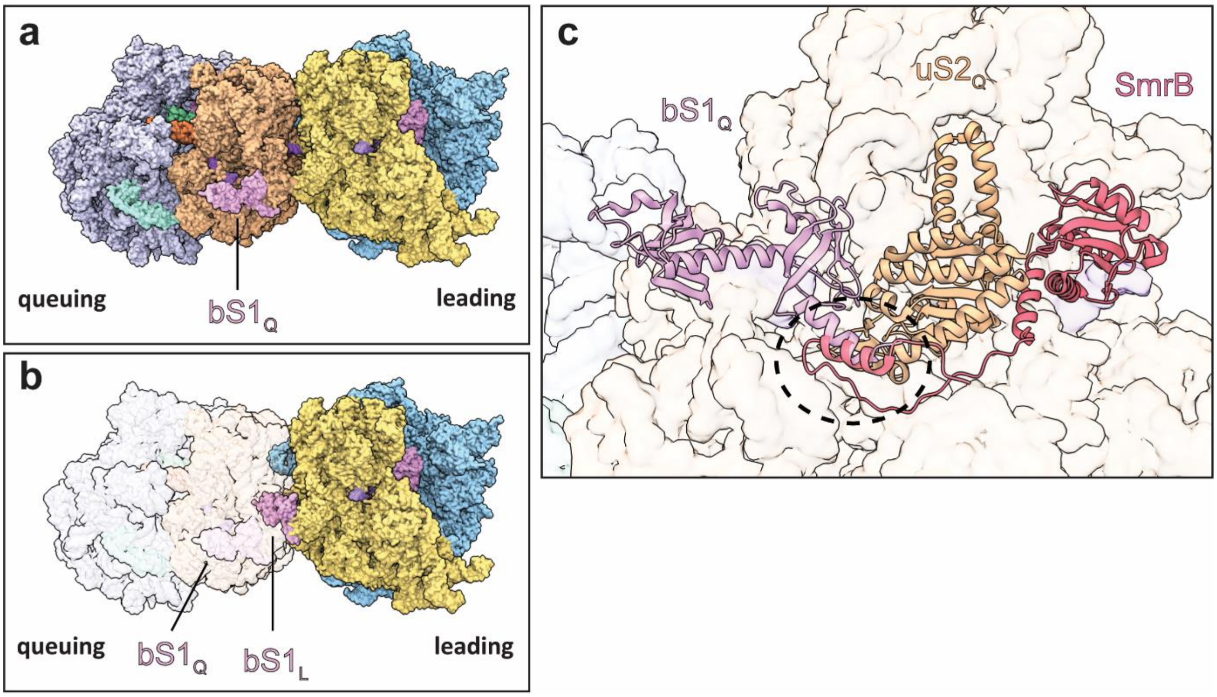

Supplementary Fig. 8: bS1$_Q$ binding site on 30S$_Q$. a and **b** superimposition of bS1 on 70S$_L$ shows steric clash with 70S$_Q$. **c** bS1$_Q$ N-terminal alpha helix (present structure) overlaps with the proposed SmrB binding site on 30S$_Q$ of the disome rescue complex (PDB: 7QGR[6]). 50S$_L$ colored in blue, 30S$_L$ in yellow, 50S$_Q$ in lavender, 30S$_Q$ in peach, A-tRNAs in yellow, P-tRNAs in green, E-tRNAs in orange, bL9$_Q$ in turquoise, and SmrB in red.

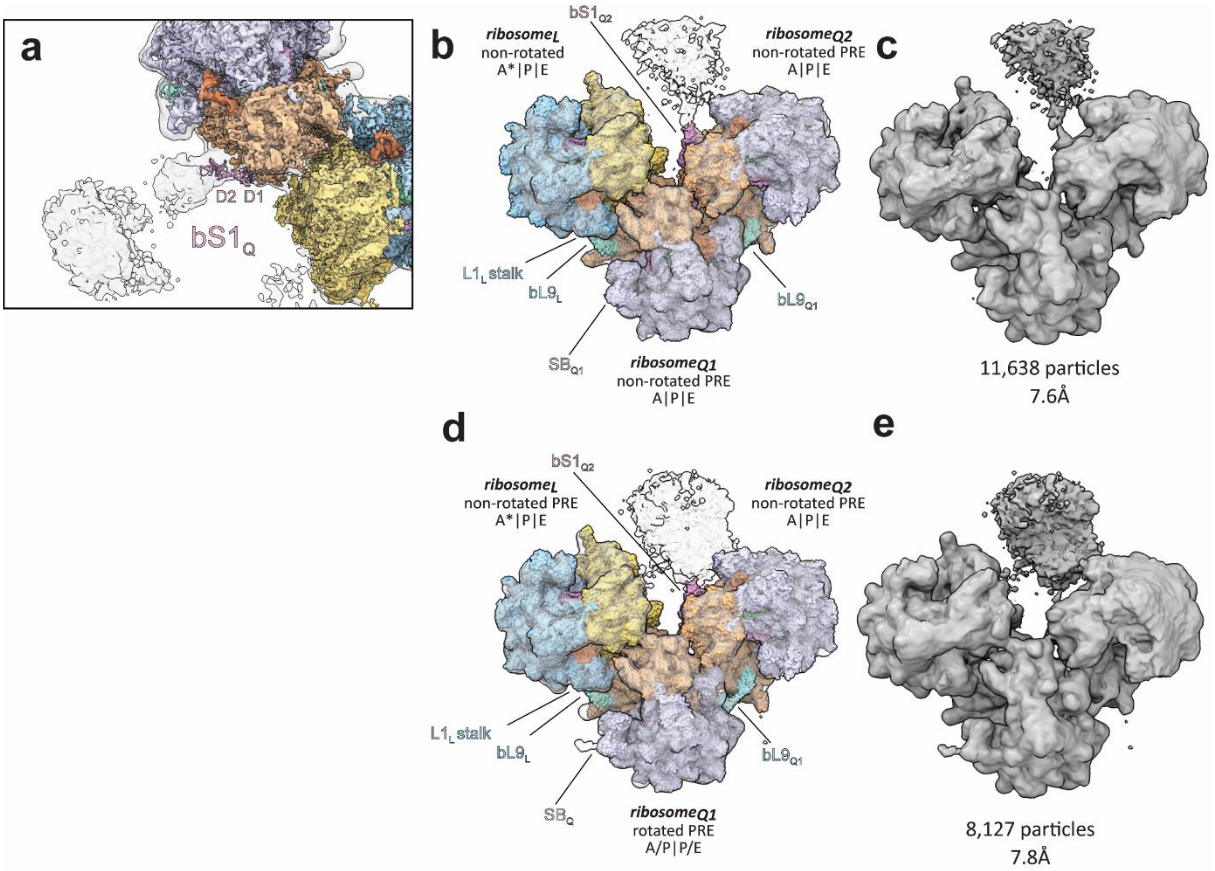

Supplementary Fig. 9: bS1$_Q$ marks the last ribosome in the queue. a AlphaFold2 generated bS1 model (P0AG67) fitted into low-pass filtered (10Å) 70S$_Q$ map. 50S$_L$ are colored in blue, 30S$_L$ in yellow, 50S$_Q$ in lavender, and 30S$_Q$ in peach. bL9$_L$ is shown in turquoise. bS1 domains (shown in pink) are abbreviated as D1-2. A-tRNAs are shown in light violet, P-tRNAs in green, and E-tRNAs in orange. **b** Non-rotated trisome complex composite model fitted into EM density (**c**). The present closed non-rotated PRE 70S$_L$ model was rigid body docked into the leading ribosome and the closed non-rotated PRE 70S$_Q$ model was rigid body docked into queueing ribosomes 1 and 2. 70S$_L$ represents a mix of non-rotated PRE and POST states. 70S$_{Q1}$ and 70S$_{Q2}$ (colors corresponding to 70S$_Q$) represent closed non-rotated PRE states with A-, P-, and E-tRNAs bound. **c** EM map of the non-rotated trisome complex. **d** Rotated trisome complex composite model fitted into EM density (**e**). The present closed non-rotated PRE 70S$_L$ model was rigid body docked into the leading ribosome, the rotated PRE state (PDB: 7N30[21]) was rigid body docked into queueing ribosome 1, and the present 70S$_Q$ model was rigid body docked into queueing ribosome 2. 70S$_L$ represents a mix of non-rotated PRE and POST states. 70S$_{Q1}$ represents a rotated-PRE state and 70S$_{Q2}$ represents a non-rotated PRE state with A-, P-, and E-tRNAs bound (colors corresponding to 70S$_Q$). **b** and **d** Stalk base is indicated as SB.

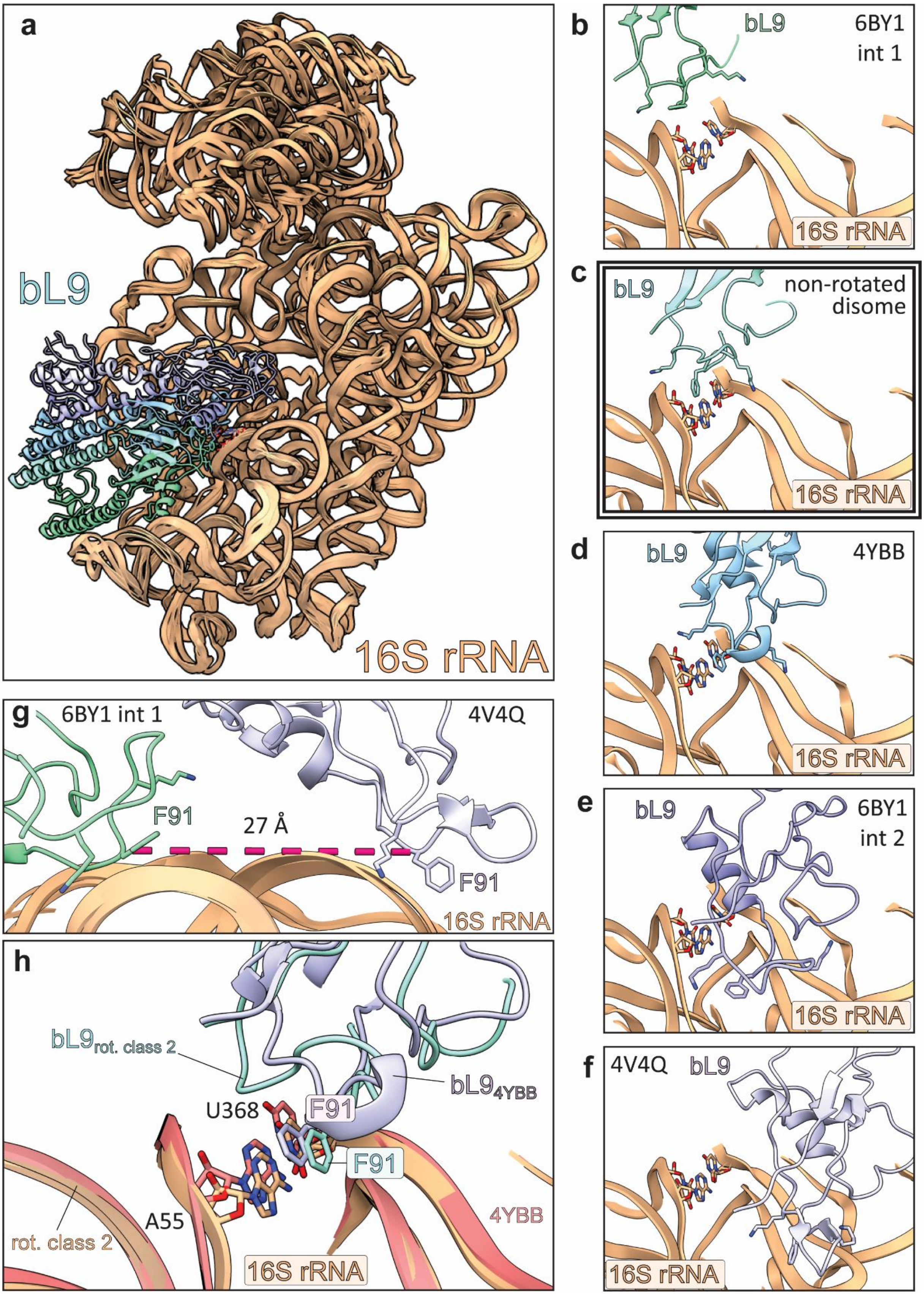

Supplementary Fig. 10: bL9$_L$ CTD and 16S rRNA$_Q$ binding plasticity. a Superimposition of the non-rotated bL9 structure bound to 30S$_Q$ and four *E. coli* crystal structures showing contacts between the bL9 CTD and the 16S rRNA of neighboring 70S in crystal packing (in the case of 6BY1[49] two

distinct interactions were observed). For structural analysis, PDB models were aligned with the 16S rRNA of the present $30S_Q$ model. **b-f** Close-ups of bL9 and 16S rRNA contacts. From top to bottom: **b** PDBs: 6BY1[49] (interaction 1), **c** present disome structure, **d** 4YBB[47], **e** 6BY1[49] (interaction 2), and **f** 4V4Q[48]. **g** Phe91 in 6BY1[49] (int1) and Phe91 in 4V4Q[48] are 27 Å apart. Distance between both Phe91 Cα was measured using the distance tool in ChimeraX[60]. **h** Overlay of rotated disome interface class 2 and 4YBB. Models were aligned with the 16S $rRNA_Q$. Shown is a close-up of the $bL9_L$:16S $rRNA_Q$ interaction illustrating the conformational similarities between both structures.

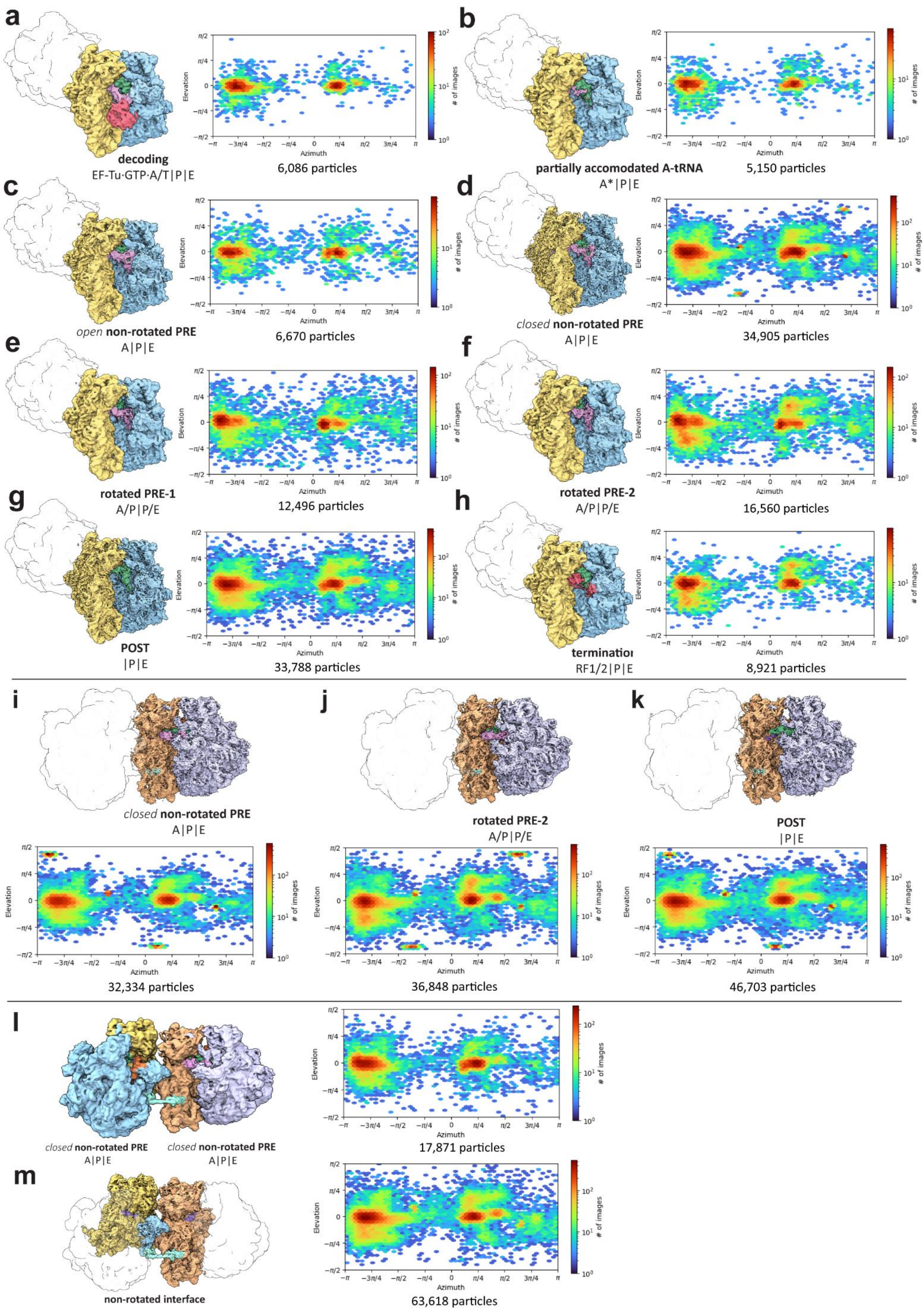

a
decoding
EF-Tu·GTP·A/T|P|E
6,086 particles
b
partially accomodated A-tRNA
A*|P|E
5,150 particles
c
open non-rotated PRE
A|P|E
6,670 particles
d
closed non-rotated PRE
A|P|E
34,905 particles
e
rotated PRE-1
A/P|P/E
12,496 particles
f
rotated PRE-2
A/P|P/E
16,560 particles
g
POST
|P|E
33,788 particles
h
terminatioı
RF1/2|P|E
8,921 particles
i
closed non-rotated PRE
A|P|E
32,334 particles
j
rotated PRE-2
A/P|P/E
36,848 particles
k
POST
|P|E
46,703 particles
l
closed non-rotated PRE
A|P|E
closed non-rotated PRE
A|P|E
17,871 particles
m
non-rotated interface
63,618 particles
Elevation
Azimuth
# of images

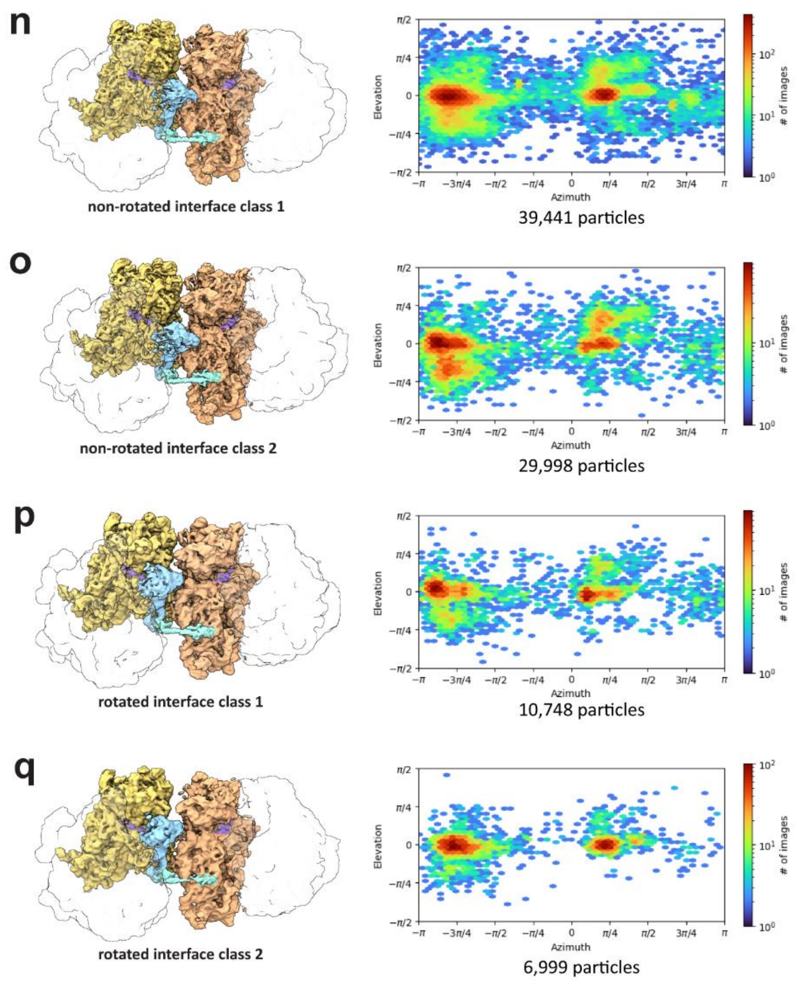

Supplementary Fig. 11: Angular distribution of final particle populations. Shown are final reconstruction of sorted functional states and disome interface classes along with angular particle distribution plots and particle numbers. **a-h** $70S_L$ functional states. **i-k** $70S_Q$ functional states. **l** Globally refined map of the disome complex containing $70S_L$ and $70S_Q$ in closed non-rotated PRE states. **m** non-rotated disome interface. **n-q** Disome interface classes. Functional states colored in yellow ($30S_L$), blue ($50S_L$), peach ($30S_Q$), lavender ($50S_Q$), light violet (A-tRNA), green (P-tRNA), orange (E-tRNA), and red (translation factors). Disome complex regions excluded by the refinement masks are indicated as silhouettes.

Supplementary Data Table 1: Cryo-EM data collection, refinement and validation statistics

	#1 *leading* 70S non-rot. *closed* PRE	#2 *queueing* 70S non-rot. *closed* PRE	#3 non-rot disome interface	#4 non-rot. interface class 1	#5 non-rot. interface class 2	#6 rot. interface class 1	#7 rot. interface class 2
**Accession numbers**	EMD-17743 PDB 8PKL	EMD-17631 PDB 8PEG	EMD-18875 PDB 8R3V	EMD-19054 PDB 8RCL	EMD-19055 PDB 8RCM	EMD-19058 PDB 8RCS	EMD-19059 PDB 8RCT
**Data collection and processing**							
Magnification	81,000x	81,000x	81,000x	81,000x	81,000x	81,000x	81,000x
Voltage (kV)	300	300	300	300	300	300	300
Electron exposure (e^{-}/Å^2)	45	45	45	45	45	45	45
Defocus range (μm)	-0.5 - -2	-0.5 - -2	-0.5 - -2	-0.5 - -2	-0.5 - -2	-0.5 - -2	-0.5 - -2
Pixel size (Å)	0.53 (1.06)	0.53 (1.06)	0.53 (1.06)	0.53 (1.59)	0.53 (1.59)	0.53 (1.59)	0.53 (1.59)
Symmetry imposed	C1	C1	C1	C1	C1	C1	C1
Initial particle images (no.)	1,883,839	1,883,839	1,883,839	1,883,839	1,883,839	1,883,839	1,883,839
Final particle images (no.)	34,905	32,334	63,618	39,441	29,998	10,748	6,999
Map resolution (Å)	3.09	3.3	3.28	3.49	3.59	4.46	5.32
FSC threshold	0.143	0.143	0.143	0.143	0.143	0.143	0.143
Map resolution range (Å)	2.3 - 30	2.3 - 30	2.3 - 30	3.2 - 30	3.2 - 30	3.2 - 30	3.2 - 30
Map sharpening B factor (Å^2)	71.9	73.4	87.2	87.2	86.0	98.8	119.3
**Refinement**							
Initial models used (PDB code)	7N1P	7N1P	7N1P	7N1P	7N1P	7N1P, 7SSN	7N1P, 7SSN
Model resolution (Å) FSC threshold = 0.143	3.08	3.73	3.28	3.50	3.61	4.55	5.35
Model resolution (Å) FSC threshold = 0.5	3.54	4.63	4.18	4.51	4.85	6.76	8.40
Model vs. map correlation coefficient (cc_mask)	0.80	0.73	0.77	0.83	0.81	0.79	0.76
Model composition							
Non-hydrogen atoms	151,740	153,292	188,616	188,663	188,203	187,326	187,388
Protein residues	6173	6329	6205	6211	6205	6315	6315
RNA residues	4810	4825	6496	6496	6478	6402	6405
Ligands/water	307	264	304	304	303	274	273
B factors (Å^2) (mean)							
Protein	106.45	197.03	132.99	170.2	171.11	250.17	378.4
RNA	118.41	176.17	165.06	212.0	221.16	332.89	410.88
Ligands	75.03	80.54	98.26	158.9	182.36	310.94	395.34
Water	64.45	-	-	-	-	-	-
R.m.s. deviations							
Bond lengths (Å)	0.004	0.006	0.002	0.002	0.007	0.003	0.003
Bond angles (°)	0.561	0.722	0.523	0.507	0.556	0.578	0.562
Validation							
MolProbity score	1.80	1.75	2.01	2.06	2.08	2.13	2.14
Clashscore	8.21	8.33	9.81	11.38	11.88	13.70	13.72
Poor rotamers (%)	0.16	0.08	0.10	0.14	0.14	0.12	0.02
Ramachandran plot							
Favored (%)	94.81	95.71	91.78	91.95	91.97	92.09	91.85
Allowed (%)	4.90	4.02	7.57	7.49	7.47	7.38	7.43
Disallowed (%)	0.30	0.27	0.66	0.56	0.56	0.53	0.73
Validation (RNA)							
Good sugar pucker (%)	98.57	98.38	98.37	98.45	98.32	98.48	98.44
Good backbone (%)	80.81	83.01	78.00	78.09	78.08	78.21	78.07

Accession numbers	#8 *leading* 70S decoding EMD-19094	#9 *leading* 70S part. accom. A-tRNA EMD-19095	#10 *leading* 70S *open* non-rot. PRE EMD-19098	#11 *leading* 70S rot. PRE-1 EMD-19096	#12 *leading* 70S rot. PRE-2 EMD-19097	#13 *leading* 70S POST EMD-19104	#14 *leading* 70S termination EMD-19099	#15 *queueing* 70S rot. PRE-2 EMD-19100
**Data collection and processing**								
Magnification	81,000x	81,000x	81,000x	81,000x	81,000x	81,000x	81,000x	81,000x
Voltage (kV)	300	300	300	300	300	300	300	300
Electron exposure (e–/Å^2)	45	45	45	45	45	45	45	45
Defocus range (μm)	-0.5 - -2	-0.5 - -2	-0.5 - -2	-0.5 - -2	-0.5 - -2	-0.5 - -2	-0.5 - -2	-0.5 - -2
Pixel size (Å)	0.53 (1.59)	0.53 (1.59)	0.53 (1.59)	0.53 (1.59)	0.53 (1.59)	0.53 (1.59)	0.53 (1.59)	0.53 (1.59)
Symmetry imposed	C1	C1	C1	C1	C1	C1	C1	C1
Initial particle images (no.)	1,883,839	1,883,839	1,883,839	1,883,839	1,883,839	1,883,839	1,883,839	1,883,839
Final particle images (no.)	6,086	5,150	6,670	12,496	16,560	33,788	8,921	36,848
Map resolution (Å)	4.61	4.56	3.79	3.45	3.22	3.21	3.82	3.21
FSC threshold	0.143	0.143	0.143	0.143	0.143	0.143	0.143	0.143
Map resolution range (Å)	3.2 - 30	3.2 - 30	3.2 - 30	3.2 - 30	3.2 - 30	3.2 - 30	3.2 - 30	3.2 - 30

Accession numbers	#16 *queueing* 70S POST EMD-19103	#17 decoding | *closed* non-rot. PRE EMD-17275	#18 decoding | rot. PRE EMD-17274	#19 decoding | POST EMD-17276	#20 part. accom. A-tRNA | *closed* non-rot. PRE EMD-17277	#21 part. accom. A-t-tRNA | rot. PRE EMD-17278	#22 part. accom. A-t-tRNA | POST EMD-17279	#23 *open* non-rot. PRE | *closed* non-rot. PRE EMD-17280
**Data collection and processing**								
Magnification	81,000x	81,000x	81,000x	81,000x	81,000x	81,000x	81,000x	81,000x
Voltage (kV)	300	300	300	300	300	300	300	300
Electron exposure (e–/Å^2)	45	45	45	45	45	45	45	45
Defocus range (μm)	-0.5 - -2	-0.5 - -2	-0.5 - -2	-0.5 - -2	-0.5 - -2	-0.5 - -2	-0.5 - -2	-0.5 - -2
Pixel size (Å)	0.53 (1.59)	0.53 (3.18)	0.53 (3.18)	0.53 (3.18)	0.53 (3.18)	0.53 (3.18)	0.53 (3.18)	0.53 (3.18)
Symmetry imposed	C1	C1	C1	C1	C1	C1	C1	C1
Initial particle images (no.)	1,883,839	1,883,839	1,883,839	1,883,839	1,883,839	1,883,839	1,883,839	1,883,839
Final particle images (no.)	46,703	2,472	1,541	2,089	1,861	1,298	1,824	2,337
Map resolution (Å)	3.21	8.18	9.06	9.11	7.9	8.93	8.41	6.95
FSC threshold	0.143	0.143	0.143	0.143	0.143	0.143	0.143	0.143
Map resolution range (Å)	3.2 - 30	6.5 - 30	6.5 - 30	6.5 - 30	6.5 - 30	6.5 - 30	6.5 - 30	6.5 - 30

Accession numbers	#24 *open* non-rot. PRE | rot. PRE EMD-17281	#25 *open* non-rot. PRE | POST EMD-17282	#26 *closed* non-rot. PRE | *closed* non-rot. PRE EMD-17283	#27 *closed* non-rot. PRE | rot. PRE EMD-17284	#28 *closed* non-rot. PRE | POST EMD-17285	#29 rotated-PRE-1 | *closed* non-rot. PRE EMD-17286	#30 rotated-PRE-1 | rot. PRE EMD-17288	#31 rotated-PRE-1 | POST EMD-17289
**Data collection and processing**								
Magnification	81,000x	81,000x	81,000x	81,000x	81,000x	81,000x	81,000x	81,000x
Voltage (kV)	300	300	300	300	300	300	300	300
Electron exposure (e–/Å^2)	45	45	45	45	45	45	45	45
Defocus range (μm)	-0.5 - -2	-0.5 - -2	-0.5 - -2	-0.5 - -2	-0.5 - -2	-0.5 - -2	-0.5 - -2	-0.5 - -2
Pixel size (Å)	0.53 (3.18)	0.53 (3.18)	0.53 (1.59)	0.53 (3.18)	0.53 (3.18)	0.53 (3.18)	0.53 (3.18)	0.53 (3.18)
Symmetry imposed	C1	C1	C1	C1	C1	C1	C1	C1
Initial particle images (no.)	1,883,839	1,883,839	1,883,839	1,883,839	1,883,839	1,883,839	1,883,839	1,883,839
Final particle images (no.)	1,763	2,587	17,871	11,961	15,613	4,884	3,829	3,824
Map resolution (Å)	7.54	7.07	4.95	6.5	6.5	8.19	8.62	8.9
FSC threshold	0.143	0.143	0.143	0.143	0.143	0.143	0.143	0.143
Map resolution range (Å)	6.5 - 30	6.5 - 30	3.2 -30	6.5 - 30	6.5 - 30	6.5 - 30	6.5 - 30	6.5 - 30

Accession numbers	#32 rotated-PRE-2 | *closed* non-rot. PRE EMD-17291	#33 rotated-PRE-2 | rot. PRE EMD-17431	#34 rotated-PRE-2 | POST EMD-17432	#35 POST | *closed* non-rot. PRE EMD-17433	#36 POST | rot. PRE EMD-17434	#37 POST | POST EMD-17441	#38 termination | *closed* non-rot. PRE EMD-17442	#39 termination | rot. PRE EMD-17443
**Data collection and processing**								
Magnification	81,000x	81,000x	81,000x	81,000x	81,000x	81,000x	81,000x	81,000x
Voltage (kV)	300	300	300	300	300	300	300	300
Electron exposure (e–/Å^2)	45	45	45	45	45	45	45	45
Defocus range (μm)	-0.5 - -2	-0.5 - -2	-0.5 - -2	-0.5 - -2	-0.5 - -2	-0.5 - -2	-0.5 - -2	-0.5 - -2
Pixel size (Å)	0.53 (3.18)	0.53 (3.18)	0.53 (3.18)	0.53 (3.18)	0.53 (3.18)	0.53 (3.18)	0.53 (3.18)	0.53 (3.18)
Symmetry imposed	C1	C1	C1	C1	C1	C1	C1	C1
Initial particle images (no.)	1,883,839	1,883,839	1,883,839	1,883,839	1,883,839	1,883,839	1,883,839	1,883,839
Final particle images (no.)	5,305	5,623	5,677	13,064	8,666	12,123	3,353	2,530
Map resolution (Å)	7.89	7.58	7.6	6.5	6.5	6.5	6.71	7.03
FSC threshold	0.143	0.143	0.143	0.143	0.143	0.143	0.143	0.143
Map resolution range (Å)	6.5 - 30	6.5 - 30	6.5 - 30	6.5 - 30	6.5 - 30	6.5 - 30	6.5 - 30	6.5 - 30

Accession numbers	#40 termination | POST EMD-17444	#41 non-rotated trisome EMD-19101	#42 rotated trisome EMD-19102
**Data collection and processing**			
Magnification	81,000x	81,000x	81,000x
Voltage (kV)	300	300	300
Electron exposure (e–/Å^2)	45	45	45
Defocus range (μm)	-0.5 - -2	-0.5 - -2	-0.5 - -2
Pixel size (Å)	0.53 (3.18)	0.53 (3.18)	0.53 (3.18)
Symmetry imposed	C1	C1	C1
Initial particle images (no.)	1,883,839	1,883,839	1,883,839
Final particle images (no.)	3,060	11,638	8,127
Map resolution (Å)	6.66	7.6	7.8
FSC threshold	0.143	0.143	0.143
Map resolution range (Å)	6.5 - 30	6.5 - 30	6.5 - 30

Supplementary Data Table 2: Inter-ribosomal distances at disome interfaces

Involved residues		*Disome interface map (this work)*			
**leading**	**queueing**	**#4**	**#5**	**#6**	**#7**
**Interface 1**		**inter-residue distance (Å)***			
**uS9 (chain ID I2)**	**uS10 (chain ID J1)**				
Y90	D85	12.4	10.5	11	17.2
D91	R89	12.6	10.4	11.1	17.6
S93	R31	9.0	10.7	9.9	12.6
E92	R31	8.5	9.6	9.4	14.1
K60	D85	10.4	11.1	10.3	13
**Interface 2**		**inter-residue distance (Å)***			
**16S rRNA (chain ID A2)**	**uS2 (chain ID B)**				
A841	K59	9.2	8.9	9.1	10.9
U843	R63	11.3	10.9	9.8	11
U843	K59	11.4	11.2	8.1	10
**uS2 (chain ID B2)**	**uS2 (chain ID B)**				
R35	E223	14.5	14.2	18.5	21.3
**Interface 3**		**inter-residue distance (Å)***			
**bS6 (chain ID F2)**	**uS4 (chain ID D1)**				
D13	R184	8.7	8.9	12.9	11.3
D13	K183	7.3	7.5	13.8	12.5
E16	N140	11.3	12.0	20.1	18.9
R44	E187	9.6	9.8	15.6	14.5
K56	E187	12.0	12.2	13.9	13.1
K56	D190	9.7	9.8	13.7	12.4
**Interface 4**		**inter-residue distance (Å)***			
**bL9 (chain ID i2)**	**16S rRNA (chain ID A1)**				
F91	U368	stack	stack	stack	stack opposite side
K89	U368	stack	stack	stack	stack
**Interface 5**		**inter-residue distance (Å)***			
**23S rRNA (chain ID 72)**	**16S rRNA (chain ID A1)**				
U2139	A412	NI	NI	stack	NI
G2152	G433	10.0	10.9	19.2	NI
**Interface 6**		**inter-residue distance (Å)***			
**uS11 (chain ID K2)**	**uS4 (chain ID D1)**				
K14	V25	12.5	12.2	7.1	6.5
Q15	R26	8.6	8.4	5.8	5.5
V16	A27	8.2	8.6	7	6
**Interface 7**		**inter-residue distance (Å)***			
**uS7(chain ID G2)**	**uS3 (chain ID C1)**				
E63	K80	14.4	14.2	10	20.5
E67	K79	15.4	15.1	11.5	16.7
**Interface 8**		**inter-residue distance (Å)***			
**uS7(chain ID G2)**	**16S rRNA (chain ID A1)**				
G55	U1030	13.6	11.4	15.7	15.5
K56	U1030	13.4	11.0	12.7	17.5

*CA-CA distances for protein:protein interactions; C1'-C1' distances for RNA:RNA interactions; CA-C1' distances for protein:RNA interactions; stack = aromatic stacking or aliphatic-aromatic stacking, NI = no interaction (distance > 20 Å)

Uncropped gel scan from Supplementary Fig. 1c

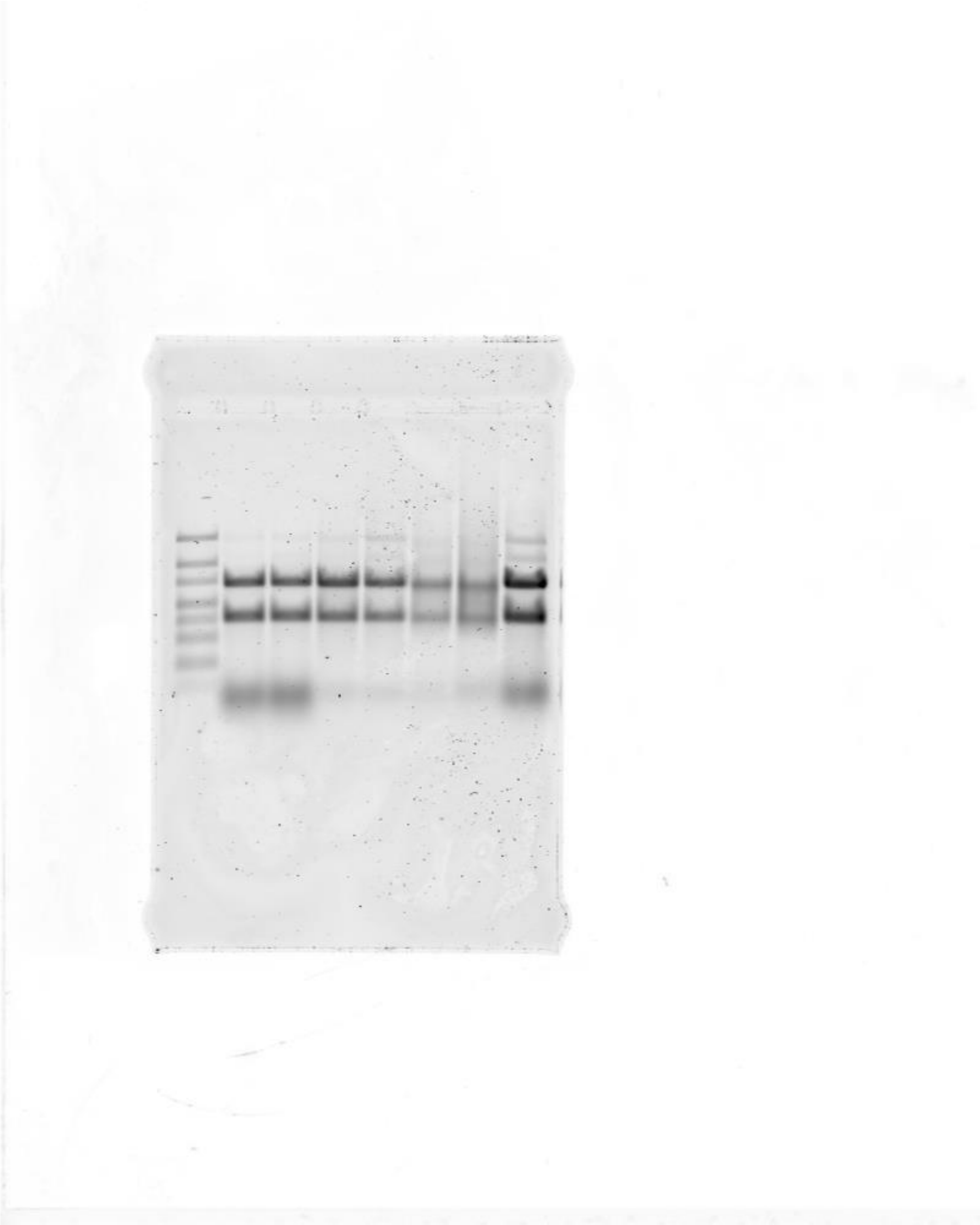

Supplement: Supplementary file 1 — Supplementary Information [file 41467_2024_46092_MOESM1_ESM.pdf]
