# Supplementary material for: Transient disome complex formation in native polysomes during ongoing protein synthesis captured by cryo-EM
Source: Nat Commun. 2024 Feb 26;15:1756. doi: 10.1038/s41467-024-46092-3 (PMC10897467; doi:10.1038/s41467-024-46092-3)
Supplement: Supplementary file 3 — Description of Additional Supplementary Files [file 41467_2024_46092_MOESM3_ESM.pdf]

File name: Supplementary Movie 1

Description: Disome interface dynamics during $70S_L$ elongation. Shown is one disome state per frame. $70S_L$ functional states are labeled in each frame. The sequence starts with the $70S_L$ decoding state and shows four rounds of elongation, finally ending with the $70S_L$ termination state. Shown are $50S_L$ (blue), $30S_L$ (yellow), $50S_Q$ (lavender), $30S_Q$ (peach), $bL9_L$ (turquoise), A-tRNAs (light violet), P-tRNAs (green), E-tRNAs (orange), and mRNA (purple).
