# Supplementary material for: Transient disome complex formation in native polysomes during ongoing protein synthesis captured by cryo-EM
Source: Nat Commun. 2024 Feb 26;15:1756. doi: 10.1038/s41467-024-46092-3 (PMC10897467; doi:10.1038/s41467-024-46092-3)
Supplement: Supplementary file 5 — Reporting Summary [file 41467_2024_46092_MOESM5_ESM.pdf]

nature portfolio

Corresponding author(s): Christian M.T. Spahn
Yollete V. Guillén Schlippe

Last updated by author(s): 2023/05/03

Reporting Summary

Nature Portfolio wishes to improve the reproducibility of the work that we publish. This form provides structure for consistency and transparency in reporting. For further information on Nature Portfolio policies, see our Editorial Policies and the Editorial Policy Checklist.

Statistics

For all statistical analyses, confirm that the following items are present in the figure legend, table legend, main text, or Methods section.

n/a	Confirmed	
☐	☒	The exact sample size (*n*) for each experimental group/condition, given as a discrete number and unit of measurement
☐	☒	A statement on whether measurements were taken from distinct samples or whether the same sample was measured repeatedly
☒	☐	The statistical test(s) used AND whether they are one- or two-sided<br>*Only common tests should be described solely by name; describe more complex techniques in the Methods section.*
☒	☐	A description of all covariates tested
☒	☐	A description of any assumptions or corrections, such as tests of normality and adjustment for multiple comparisons
☐	☒	A full description of the statistical parameters including central tendency (e.g. means) or other basic estimates (e.g. regression coefficient) AND variation (e.g. standard deviation) or associated estimates of uncertainty (e.g. confidence intervals)
☒	☐	For null hypothesis testing, the test statistic (e.g. *F*, *t*, *r*) with confidence intervals, effect sizes, degrees of freedom and *P* value noted<br>*Give P values as exact values whenever suitable.*
☒	☐	For Bayesian analysis, information on the choice of priors and Markov chain Monte Carlo settings
☒	☐	For hierarchical and complex designs, identification of the appropriate level for tests and full reporting of outcomes
☒	☐	Estimates of effect sizes (e.g. Cohen's *d*, Pearson's *r*), indicating how they were calculated

Our web collection on statistics for biologists contains articles on many of the points above.

Software and code

Policy information about availability of computer code

Data collection	Cryo-electron microscopy data collection utilized the following software/code: EPU (FEI company), Leginon (Carragher et al., 2000; Suloway et al., 2005)
Data analysis	Cryo-electron microscopy data analysis utilized the following software/code: Cryosparc v3.3.1 (Punjani et al., 2017), Coot Coot 0.9.6 (Emsley and Cowtan, 2004), ERRASER (Chou et al, 2013), Gautomatch (developed by K. Zhang), Gctf (Zhang, 2016), MotionCor2 (Zheng et al., 2017), Warp (Tegunov and Cramer, 2019), PHENIX 1.19 (Adams et al., 2010), Relion 3.0 (Zivanov et al. 2018), SPIDER (Frank et al, 1996), UCSF Chimera v1.13.1 (Petterson et al., 2004), UCSF ChimeraX 1.3 (Petterson et al., 2021), AlphaFold (Jumper et al., 2021), MolProbity (Williams et al., 2018), Sigmaplot 14.0.

For manuscripts utilizing custom algorithms or software that are central to the research but not yet described in published literature, software must be made available to editors and reviewers. We strongly encourage code deposition in a community repository (e.g. GitHub). See the Nature Portfolio guidelines for submitting code & software for further information.

Data

Policy information about availability of data

All manuscripts must include a data availability statement. This statement should provide the following information, where applicable:

- Accession codes, unique identifiers, or web links for publicly available datasets
- A description of any restrictions on data availability
- For clinical datasets or third party data, please ensure that the statement adheres to our policy

All structures presented in this manuscript have been deposited to the wwPDB and will become available upon publication.

#1 leading non-rotated closed PRE (EMD-17743; PDB 8PKL)
#2 queueing non-rotated closed PRE (EMD-17631; PDB 8PEG)
#3 non-rotated disome interface (EMD-18875; PDB 8R3V)
#4 non-rotated interface class 1 (EMD-19054; PDB 8RCL)
#5 non-rotated interface class 2 (EMD-19055; PDB 8RCM)
#6 rotated interface class 1 (EMD-19058; PDB 8RCS)
#7 rotated interface class 2 (EMD-19059; PDB 8RCT)
#8 leading 70S decoding (EMD-19094)
#9 leading 70S partially accommodated A-tRNA (EMD-19095)
#10 leading 70S open non-rotated PRE (EMD-19098)
#11 leading 70S rotated PRE-1 (EMD-19096)
#12 leading 70S rotated PRE-2 (EMD-19097)
#13 leading 70S POST (EMD-19104)
#14 leading 70S termination (EMD-19099)
#15 queueing 70S rotated PRE-2 (EMD-19100)
#16 queueing 70S POST (EMD-19103)
#17 decoding | closed non-rotated PRE (EMD-19103)
#18 decoding | rotated PRE-2 (EMD-17274)
#19 decoding | POST (EMD-17276)
#20 partially accommodated A-tRNA | closed non-rotated PRE (EMD-17277)
#21 partially accommodated A-t-tRNA | rotated PRE-2 (EMD-17278)
#22 partially accommodated A-t-tRNA | POST (EMD-17279)
#23 open non-rotated PRE | closed non-rotated PRE (EMD-17280)
#24 open non-rotated PRE | rotated PRE-2 (EMD-17281)
#25 open non-rotated PRE | POST (EMD-17282)
#26 closed non-rotated PRE | closed non-rotated PRE (EMD-17283)
#27 closed non-rotated PRE | rotated PRE-2 (EMD-17284)
#28 closed non-rotated PRE | POST (EMD-17285)
#29 rotated PRE-1 | closed non-rotated PRE (EMD-17286)
#30 rotated PRE-1 | rotated PRE-2 (EMD-17288)
#31 rotated PRE-1 | POST (EMD-17289)
#32 rotated PRE-2 | closed non-rotated PRE (EMD-17291)
#33 rotated PRE-2 | rotated PRE-2 (EMD-17431)
#34 rotated PRE-2 | POST (EMD-17432)
#35 POST | closed non-rotated PRE (EMD-17433)
#36 POST | rotated PRE-2 (EMD-17434)
#37 POST | POST (EMD-17441)
#38 termination | closed non-rotated PRE (EMD-17442)
#39 termination | rotated PRE-2 (EMD-17443)
#40 termination | POST (EMD-17444)
#41 non-rotated trisome (EMD-19101)
#42 rotated trisome (EMD-19102)

Research involving human participants, their data, or biological material

Policy information about studies with human participants or human data. See also policy information about sex, gender (identity/presentation), and sexual orientation and race, ethnicity and racism.

Reporting on sex and gender	N/A
Reporting on race, ethnicity, or other socially relevant groupings	N/A
Population characteristics	N/A
Recruitment	N/A
Ethics oversight	N/A

Note that full information on the approval of the study protocol must also be provided in the manuscript.

Field-specific reporting

Please select the one below that is the best fit for your research. If you are not sure, read the appropriate sections before making your selection.

☒ Life sciences ☐ Behavioural & social sciences ☐ Ecological, evolutionary & environmental sciences

For a reference copy of the document with all sections, see nature.com/documents/nr-reporting-summary-flat.pdf

Life sciences study design

All studies must disclose on these points even when the disclosure is negative.

Sample size	For cryo-EM the number of particle images was limited by the available beam time.
Data exclusions	Cryo-EM reconstructions corresponding to contaminations (e.g. ice) were removed from the analysis, as described in the sorting scheme (supplementary Fig. 2).
Replication	Translation activity assays were performed at least in duplicates. Several batches of polysomal samples were prepared by size exclusion gel filtration. One batch of polysomes was used for analysis of the restimulated polysomes (kinetic assays and cryo-EM). Another batch was used for cryo-EM analysis of the non-restimulated polysomes.
Randomization	Not relevant for this study.
Blinding	Not relevant for this study.

Reporting for specific materials, systems and methods

We require information from authors about some types of materials, experimental systems and methods used in many studies. Here, indicate whether each material, system or method listed is relevant to your study. If you are not sure if a list item applies to your research, read the appropriate section before selecting a response.

Materials & experimental systems

n/a	Involved in the study
☒	☐ Antibodies
☒	☐ Eukaryotic cell lines
☒	☐ Palaeontology and archaeology
☒	☐ Animals and other organisms
☒	☐ Clinical data
☒	☐ Dual use research of concern
☒	☐ Plants

Methods

n/a	Involved in the study
☒	☐ ChIP-seq
☒	☐ Flow cytometry
☒	☐ MRI-based neuroimaging
